# A scoping review of digital health applications for managing noncommunicable diseases in primary care post-pandemic: Lessons from the Western Pacific Region

**DOI:** 10.51866/rv.919

**Published:** 2025-12-02

**Authors:** Muhammad Solehuddin Ishak, Rizq Fazzali Abdul Raes, Ihsan Abdul Razak, Mohd Hanif Mohd Nawawi, Suhana Hasan, Afiq Izzudin A Rahim

**Affiliations:** 1 MB BCH BAO, MPH DrPH, Department of Community Medicine, School of Medical Science, Health Campus, Universiti Sains Malaysia, Kubang Kerian, Kelantan, Malaysia. E-mail: drafiqrahim@usm.my; 2 MBBS, MPH, Department of Community Medicine, School of Medical Science, Health Campus, Universiti Sains Malaysia, Kubang Kerian, Kelantan, Malaysia.; 3 MBBS, MPH, Department of Community Medicine, School of Medical Science, Health Campus, Universiti Sains Malaysia, Kubang Kerian, Kelantan, Malaysia.; 4 MBBS, MPH, Department of Community Medicine, School of Medical Science, Health Campus, Universiti Sains Malaysia, Kubang Kerian, Kelantan, Malaysia.; 5 MBBCh, MPH, Department of Community Medicine, School of Medical Science, Health Campus, Universiti Sains Malaysia, Kubang Kerian, Kelantan, Malaysia.; 6 MBBS, MPH, Department of Community Medicine, School of Medical Science, Health Campus, Universiti Sains Malaysia, Kubang Kerian, Kelantan, Malaysia.

**Keywords:** Digital health, Primary health care, Noncommunicable disease, Pacific islands

## Abstract

**Introduction:**

The global rise of digital health is reshaping healthcare delivery and improving outcomes, especially for noncommunicable diseases (NCDs) post-pandemic. Guided by the World Health Organization (WHO) Global Strategy on Digital Health 2020-2025, this study examined digital health applications in Western Pacific Region (WPR) primary care, focusing on NCD management and related challenges.

**Methods:**

A scoping review following the Arksey and O’Malley framework and Preferred Reporting Items for Systematic Reviews and Meta-Analyses Extension for Scoping Reviews guidelines was conducted. Using the PICO framework, studies on digital interventions in WPR primary care were identified through PubMed, Scopus, ScienceDirect, Web of Science and the *Journal of Medical Internet Research* (2022-2024). Eighteen studies were synthesized using the WHO digital health intervention and Health System Challenge frameworks.

**Results:**

The review included studies from Australia (n=10), Singapore (n=5), South Korea (n=1), and China (n=2), encompassing randomized, observational, qualitative, and pragmatic designs. Digital interventions—telehealth, mobile health (mHealth) and electronic health (eHealth)—targeted NCDs such as diabetes, mental health, and cardiovascular diseases, addressing information quality, acceptability, efficiency, cost, accountability, and equity.

**Conclusion:**

The scoping review identified several digital health interventions, predominantly telehealth, mHealth and eHealth, deployed across Australia, Singapore, South Korea and China for NCD management in primary care. The studies demonstrated improvements in information quality, acceptability and efficiency, while highlighting persistent barriers such as technology integration issues, data quality concerns and inequities.

## Introduction

The rapid advancement of information and communication technologies (ICTs) has significantly influenced service delivery across various industries, including healthcare. The integration of ICTs in healthcare, commonly referred to as digital health, has become a major global initiative supported by organisations such as the World Health Organization (WHO).^1^ The WHO Global Strategy on Digital Health 2020-2025 highlights the importance of adopting and scaling up digital health innovations to enhance health outcomes and strengthen health systems in pursuit of universal health coverage.^2,3^

Digital health encompasses a broad spectrum of technologies, including mobile health (mHealth), telehealth, electronic health (eHealth), wearable devices and artificial intelligence (AI)-based healthcare solutions. These technologies play a crucial role in improving disease management, remote patient monitoring, clinical decision-making and patient-centred care, especially within primary health care (PHC) settings.^3,4^ Given that PHC serves as the first point of contact for patients and provides accessible, continuous and comprehensive care, integrating digital health into primary care has the potential to revolutionise healthcare delivery, particularly for the management of noncommunicable diseases (NCDs).^4-6^

NCDs such as diabetes, cardiovascular diseases and hypertension pose significant health challenges worldwide. The burden of these chronic conditions has been exacerbated by rapid urbanisation and lifestyle changes, particularly in the Western Pacific Region (WPR), where urban populations are growing rapidly.^7,8^ The COVID-19 pandemic further strained healthcare systems, making digital health adoption a necessary solution for addressing both existing and emerging healthcare challenges.^9^ During the pandemic, digital health interventions (DHIs), such as telemedicine, mHealth applications and remote patient monitoring, became critical tools in managing NCDs while minimising physical contact between patients and healthcare providers.^3,7,8^

Despite the recognised potential of digital health, its implementation in PHC settings within the WPR remains underexplored and fragmented. There is a lack of comprehensive reviews that assess the effectiveness, challenges and best practices of digital health applications for NCD management in this region.^2,4,10^ This scoping review aimed to fill this gap by systematically mapping the use of digital health in PHC settings across the WPR, evaluating its effectiveness in managing NCDs and identifying common barriers and challenges to its adoption.

Given that Malaysia is part of the WPR, the insights gained from this review will provide evidence-based recommendations to enhance the country’s digital health strategies, ensuring that digital interventions are effectively integrated into primary care. By leveraging lessons from other WPR nations, Malaysia can optimise its digital health ecosystem to improve NCD management and strengthen its healthcare system in the post-pandemic era.^10^

## Methods

This scoping review followed the Arksey and O’Malley framework, consisting of five key steps: (1) defining research questions, (2) identifying relevant literature, (3) selecting publications, (4) extracting data and (5) analysing data and summarising and reporting results.^11,12^ The Preferred Reporting Items for Systematic Reviews and Meta-Analyses Extension for Scoping Reviews (PRISMA-ScR) checklist was used to ensure comprehensive reporting.^13^

### Research questions

Three primary research questions guided this review:

1. What is the current landscape of digital health applications for managing NCDs in primary care across the WPR post-pandemic?
2. What types of digital health applications are most frequently used for NCD management in primary care?
3. What are the barriers and challenges to implementing digital health applications for NCD management in the post-pandemic era?

### Identification of relevant studies

The PICO framework was employed to establish eligibility criteria and guide the search strategy.^14^ The inclusion criteria covered primary studies (randomised controlled trials [RCTs] or quasiexperimental studies) examining DHIs for NCD management in primary care settings. Studies conducted outside the WPR, those in hospital settings, those conducted before or during the height of the COVID-19 pandemic (before 2022), non-English papers and review articles were excluded.

### Search strategy and databases

A systematic search was conducted across five databases - PubMed, ScienceDirect, Scopus, Web of Science and the *Journal of Medical Internet Research* (JMIR) - using customised Boolean operators to ensure relevance. The search strategy incorporated key terms related to digital health (e.g. ‘digital health’ OR ‘eHealth’ OR ‘mHealth’), NCDs (e.g. ‘NCDs’ OR ‘chronic diseases’ OR ‘diabetes’) and PHC (e.g. ‘primary care’ OR ‘clinic’), along with country-specific terms for the WPR. For example, in PubMed, the search query included the following: (‘digital health’ OR ‘digital intervention’ OR ‘digital interventions’ OR ‘e health’ OR ‘eHealth’ OR ‘mHealth’ OR ‘m health’ OR ‘mobile health’ OR ‘mobile phone’ OR ‘telehealth’ OR ‘telemedicine’ OR ‘telemonitoring’ OR ‘video consultation’ OR ‘virtual care’ OR ‘virtual clinic’) AND (‘primary care’ OR ‘primary healthcare’ OR ‘clinic’ OR ‘GP’ OR ‘general practice’) AND (‘NCDs’ OR ‘NCD’ OR ‘chronic diseases’ OR ‘diabetes’ OR ‘hypertension’ OR ‘cardiovascular’) AND (‘Western Pacific Region’ OR ‘western pacific country’ OR ‘Australia’ OR ‘Brunei’ OR ‘Cambodia’ OR ‘China’ OR ‘Cook Islands’ OR ‘Fiji’ OR ‘Japan’ OR ‘Kiribati’ OR ‘Laos’ OR ‘Malaysia’ OR ‘Marshall Islands’ OR ‘Micronesia’ OR ‘Mongolia’ OR ‘Nauru’ OR ‘New Zealand’ OR ‘Niue’ OR ‘Palau’ OR ‘Papua New Guinea’ OR ‘Philippines’ OR ‘Samoa’ OR ‘Singapore’ OR ‘Solomon Islands’ OR ‘South Korea’ OR ‘Taiwan’ OR ‘Tonga’ OR ‘Tuvalu’ OR ‘Vanuatu’ OR ‘Vietnam’), retrieving 616 results. Other databases yielded 690 additional records.

### Selection and handling of studies

Reference management was handled using EndNote 20 to remove duplicates and streamline selection. The study selection followed a two-step process: (1) an initial abstract screening and (2) a fUll-text evaluation based on the predefined eligibility criteria. All five reviewers first agreed on the criteria using a small pilot set. Two reviewers then checked each title/abstract and full text; when they disagreed, a third judged, and, if needed, a fourth decided. Two reviewers extracted data from the included papers, with a fifth breaking any remaining ties.

### Data extraction and charting

Excel spreadsheets were specifically designed to align with all research questions, allowing for the synthesis of important themes. Additionally, these spreadsheets were structured to assign codes where relevant, serving as a supportive framework for subsequent analyses.

### Analysis, synthesis and reporting

In line with recommendations for standardising scoping reviews, 115 our approach followed three main steps: data analysis, results reporting and data interpretation. Wile employed a six-step iterative process for thematic content analysis and synthesis, which included data familiarisation, preliminary coding, theme exploration, theme review, theme finaiisation and report generation. The resuits were synthesised according to the PRISMA-ScR guidelines and presented using tables and thematic narratives.

### Study frameworks

Two established frameworks were utilised for categorisation and interpretation:

1. WHO digital health classification 16: DHIs were classified into four user-based categories: (1) clients (patients with NCDs), (2) healthcare providers (primary care practitioners), (3) health system managers and (4) data services (data management and exchange).
2. Health System Challenge framework: This framework was applied to categorise the barriers and challenges faced in digital health implementation for NCD management. Nine dimensions were assessed: (1) information, (2) availability, (3) quality, (4) acceptability, (5) utilisation, (6) efficiency, (7) cost, (8) accountability and (9) equity.

## Results

### Search outcome and overview of the included studies

**Figure 1:**
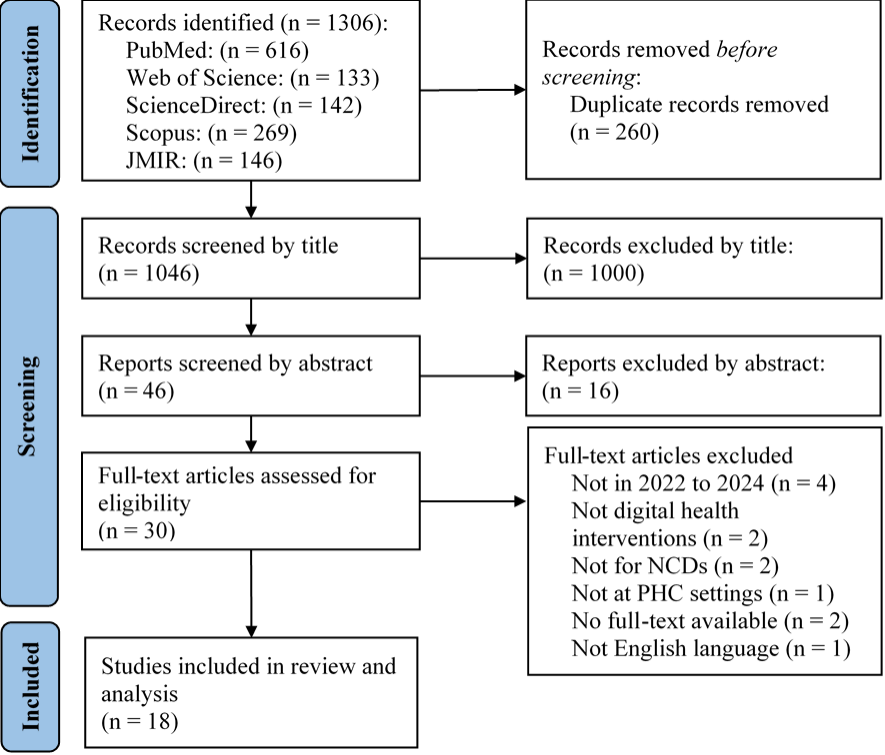
PRISMA flow diagram for the included studies.

The database and journal searches yielded 1306 records (PubMed: n=616, Web of Science: n=133, ScienceDirect: n=142, Scopus: n=269 and JMIR: n=146). After 260 duplicates were removed, 1046 ar ticles were screened based on their title and abstract, with 1016 excluded. A total of 30 full-text articles were assessed for eligibility, and 12 were excluded, resulting in 18 studies included in the final review. The reference lists of these studies ‘were also checked for additional relevant literature, as detailed in Figure 1.

### Study characteristics

The included studies, published from 2022 to 2024, focused on DHIs for NCD management in PHC settings across four WPR countries. Sixteen studies were conducted in high-income countries: Australia (n=10), Singapore (n=5) and South Korea (n=1), while two were from an upper-middle-income country: China (n=2). No Malaysian studies were found, likely due to strict criteria such as date limits, English-only publications and peer-reviewed sources, which may have excluded local or unpublished research.

A variety of research designs were used, including RCTs, observational cohort studies, feasibility trials, qualitative studies and mixed-method approaches. Table 1 provides a summary of the study characteristics, key outcomes, challenges, barriers and future recommendations.

**Table 1:** Summary of evidence from the scoping review.

Study (author and year)	Country	Study design	Study purpose	Study method	Outcome	Challenge/barrier	Recommendation
Ward et al., 2023.^16^	Australia	Secondary analysis	The study assessed GP consultations of patients with chronic illness, analysing frequency, duration, physical examinations, clinical tasks and telehealth feasibility.	Using content analysis, video review and time analysis, the researchers graded telehealth translatability.	Telehealth-translatable tasks averaged 7/10 (score 1: not amenable to replication at this point, score 10: easily translatable with practically minimal additional equipment).	A key barrier was the small sample size (38 consultations), limiting observations.	Although the study covered major chronic conditions, further research is needed to assess all conditions and enhance telehealth applicability for physical examinations and clinical tasks.
Cross et al., 2022.^17^	Australia	Prospective uncontrolled observational cohort study	This study examined the impact of patient characteristics, particularly self-reported psychosocial difficulties, on treatment uptake, completion and improvement using national DMHS data.	An online test assessed applicants, with some starting treatment and a subset completing at least four of five lectures.	Classification algorithms found that older age, higher educational level and the presence of a relationship positively influenced outcomes, while higher symptom severity had a negative effect.	Psychosocial difficulties may increase burden, reducing treatment engagement.	Future research should address burden, capacity and maintenance, and tailored, effort-optimised interventions are needed to enhance accessibility, completion rates and treatment effectiveness.
Champion et al., 2023.^18^	Australia	Cluster-randomised controlled trial	This study evaluated the efficacy of the eHealth intervention Health4Life in modifying six lifestyle risk behaviours (alcohol use, smoking, screen time, inactivity, poor diet and poor sleep) among teenagers.	Schools were randomly assigned to Health4Life or standard health education.	Primary outcomes were assessed via self-reported surveys at 24 months.	Despite widespread smartphone use among Australian teens, many did not engage with health applications unless actively seeking to change health behaviours.	Future research should optimise web- and application-based interventions by refining timing, parental involvement, motivation and interaction for adolescent health behavioural changes.
**Legends:** GP = general practitioners; DMHS = Digital mental health services; eHealth = electronic health							
Imai et al., 2022.^19^	Australia	Exploratory study using electronic patient data from a retrospective cohort study	This study evaluated telehealth uptake in Australian general practice and its impact on HbA1c testing for patients with diabetes.	Using electronic data from 800 clinics in Victoria and NSW, the researchers compared telehealth users and non-users.	During the pandemic, 80.8% of 57,916 patients used telehealth, uptake was higher among older adults, women, CKD, and antidiabetic users. HbAlc testing rates and levels remained comparable.	Limitations included not distinguishing phone versus video consultations.	Further research should explore patient preferences, improve video consultation uptake and assess long-term integration of telehealth in diabetes care.
Buss et al., 2022.^20^	Australia	Single-group feasibility trial	This study tested the feasibility of an application-based intervention for cardiovascular and diabetes risk awareness over 3 months by tracking non-usage, dropout, adherence and usability.	Participants received installation manuals, and surveys including the Mobile App Rating Scale were conducted post-study.	Out of 46 volunteers, 20 never used the application, and 15 dropped out, with only eight (26% of users) using it weekly; the application scored an average of 3.5/5, being informative but unengaging.	Barriers included limited early support, no direct healthcare contact and low interactivity.	Recommendations include early application downloads, enhanced user engagement, voice input and doctor visits.
Yin et al., 2022.^21^	China	Single-centre randomised control study	This study assessed telemedicine management for overweight and obese patients with T2DM during the COVID-19 pandemic.	Patients were randomised to telemedicine or outpatient care, received diet and exercise guidance and were monitored for 6 months.	Among 99 patients completing follow-up, telemedicine users had improved HbA1c, lipid and anthropometric markers, while the control group had poorer glycaemic control and cardiovascular risk factors.	Limitations included a single-centre design, reliance on tech-savvy patients and incomplete follow-up data.	Recommendations include expanding to multiple centres, including patients with varied monitoring needs and strengthening follow-up procedures to improve retention and data completeness.
**Legends:** HbA1c = glycated haemoglobin; NSW = New South Wales; CKD = chronic kidney disease; T2DM = type 2 diabetes mellitus; COVID-19 = coronavirus disease-2019							
Koh et al., 2023.^22^	Singapore	Retrospective cohort study	This study assessed the efficacy of telephone consultations for diabetes management compared to in-person visits.	Electronic medical records from April to September 2020 were analysed, measuring HbAlc changes pre- and post-intervention.	Statistical analysis showed that phone consultations were less effective for patients with baseline HbA1c levels of >9%, while older, less tech-savvy patients preferred them.	Limitations included selection bias and retrospective data. The findings suggest that phone consultations may not ensure long-term glucose control.	Future research should explore long-term effectiveness, patient satisfaction and cost-effectiveness, while video consultations could improve rapport despite potential technological challenges for some patients.
Tan et al., 2023.^23^	Singapore	Qualitative study	This study explored the perspectives of Singaporean patients who had not used video consultations (VCs) for NCD management.	Thematic analysis, based on the Health Information Technology Acceptance Model, identified benefits, challenges and concerns.	Patients viewed VCs as safe and convenient for stable NCDs but were worried about limited physical examinations, connectivity issues and data security.	Limitations included the inclusion of only English-speaking participants and a lack of direct VC experience.	The findings may not apply to conditions requiring physical examinations, such as asthma and COPD. Future research should include respiratory NCDs and explore experiences of VC users and healthcare providers.
**Legends:** HbAlc = glycated haemoglobin; VC = video consultations; NCD = noncommunicable disease; COPD = chronic obstructive pulmonary disease							
Wang et al., 2023.^24^	Australia	Randomised controlled trial	This study analysed in-person GP consultations of patients with chronic disease, focusing on frequency, length, physical examinations, clinical tasks and telehealth transferability.	Office and ambulatory BP readings were compared, and telemedicine feasibility was assessed through interviews.	The findings showed that older age and forgetfulness hindered home BP monitoring, while non-adherence to ambulatory BP monitoring affected result interpretation. Office BP remained similar between groups.	Limitations included excluding patients with complex comorbidities needing frequent visits.	Future research should explore telemedicine’s impact on additional stakeholders, including patient-carers and policymakers, to improve service accessibility and effectiveness.
Teo et al., 2023.^25^	Singapore	Qualitative study	This study examined the perceptions of patients and healthcare workers regarding a technology-enabled blood pressure monitoring intervention with teleconsultation in Singapore’s primary care.	Data from audio-recorded interviews were analysed to identify themes based on the sociotechnical systems model.	Participants found the intervention simple and beneficial for patient-provider relationships, as it allowed self-management and increased support. Healthcare workers appreciated the time saved for other tasks.	The findings may not apply to other settings due to differences in operational methods.	The study suggests that effective interventions can improve healthcare delivery and patient safety and reduce unnecessary hospital visits.
**Legends:** GP = general practitioners; BP = blood pressure							
Andrews et al., 2023.^26^	Australia	Adaptive randomised controlled trial	This study evaluated the efficacy of the digital mental health intervention Life Flex across three treatment conditions	DMH alone, DMH with low-intensity therapist help and DMH with high-intensity therapist support Participants were assessed at baseline and 3, 6, 9 and 21 weeks post-intervention.	Primary outcomes: GAD-7 and PHQ-9 scores. Secondary outcomes: motivation, self-efficacy, working alliance, health status, usability, satisfaction, healthcare use, preferences and diagnosis.	As the study was conducted during the COVID-19 pandemic, challenges included participant self-selection for therapist assistance and heightened anxiety or depression symptoms.	Future research should replicate and further analyse the findings for additional insight
Drabarek et al., 2022.^27^	Australia	Qualitative study	This study assessed patient-led surveillance perceptions and experiences in the 6-month MEL-SELF pilot RCT.	atients with localised melanoma in New South Wales, Australia, were randomised to use self-monitoring tools, including mobile dermatoscopes and a smartphone application for teledermatologist review.	Thematic analysis using the Technology Acceptance Model showed that higher adherence led to increased skin awareness, improved self-examination, early melanoma detection and proactive clinical follow-up.	Barriers included difficulties in obtaining clear images and technical issues, although these were overcome with prior digital experience and help from skin check partners.	The perceived usefulness varied with the number of moles.
**Legends:** DMH = Digital mental health; GAD-7 = 7-item Generalized Anxiety Disorder Scale; PHQ-9 = 9-item Patient Health Questionnaire; COVID-19 = coronavirus disease-2019; MEL-SELF = melanoma self-examinations; RCT = randomized controlled trial							
Chew et al., 2023.^28^	Singapore	Secondary qualitative analysis	This study explored patient and healthcare staff experiences with remote blood pressure monitoring, focusing on confidence in technology and patient-provider interactions.	Patients used a Bluetooth-enabled BP monitor and a mobile data gateway to upload readings weekly for 6-12 months.	Patients and staff trusted the system, improving relationships, although doctors had concerns about digital interactions and institutional risks.	Selectivity bias may have influenced the findings, and younger patients with hypertension need further study.	Telemedicine can enhance remote care by strengthening patient-provider relationships and maintaining treatment quality while ensuring efficiency through well-designed telehealth interactions that foster provider presence and trust.
Parker et al., 2022.^29^	Australia	Pragmatic two-arm cluster randomised controlled trial	This study evaluated a multifaceted intervention on diet, physical activity and health literacy in overweight and obese primary care patients.	The intervention included a nurse-led health check, the use of a mobile application and telephone coaching.	Health, weight, waist circumference, blood pressure and literacy were assessed. Secondary outcomes included diet, exercise, counselling, lipid level, quality of life and costs.	The study was limited to two Australian metropolitan areas.	Early engagement using codesign strategies may improve outcomes, especially for lower socioeconomic groups, and enhance intervention effectiveness.
**Legends:** BP = blood pressure							
Ju et al., 2022.^30^	South Korea	Pilot multicentre real-world study	This study assessed the effectiveness of combining a mobile self-management health application with human coaching for patients with chronic disease in primary care.	Participants (n=65) used the application for 12 weeks, while controls (n=45) followed traditional self-management.	The results showed that application users experienced greater weight loss and improved sleep quality than controls.	The study’s non-randomised design and small sample size limit generalisability. Age, sex, education and underlying illnesses were adjusted using propensity score matching to reduce bias.	The research highlights the potential of mobile health applications with coaching in chronic disease management.
Teo et al., 2024.^31^	Singapore	Quasi-experimental cohort study	This study assessed the clinical impact and cost-effectiveness of a home blood pressure (BP) monitor integrated into primary care.	Patients used a mobile gateway-linked BP device with nurse-led teleconsultations, and outcomes were evaluated after 6 months.	Telemonitoring improved BP management, especially in patients with uncontrolled baseline BP, with a cost-effectiveness ratio of $23,935.14/QALY.	Limitations included non-randomised participant selection, unexamined IT literacy and potential inaccuracies in self-reported BP readings.	To mitigate the identified issues, office BP was analysed instead of home-measured BP. Encouraging proper device use and ensuring accurate data submission were key considerations for clinical application.
**Legends:** BP = blood pressure; QALY = quality-adjusted life year							
Schumacher et al., 2023.^32^	Australia	Cluster randomised controlled trial	This study evaluated the feasibility, acceptability and cost-effectiveness of a telehealth medical nutrition therapy (MNT) programme for cardiovascular disease (CVD) risk reduction in rural primary care.	An accredited practising dietitian provided five telehealth consultations over 6 months, with nutrition feedback based on the AES-Heart questionnaire.	The primary outcome was total serum cholesterol reduction.	Barriers included cultural and technological challenges, with some perceiving telehealth as lower-quality care.	Prior research has shown positive experiences with telehealth for rural allied health services.
Wu et al., 2023.^33^	China	Nested mixed-method study	To evaluate the effectiveness of an AI-HEALS-based intervention on T2DM self-management and glycaemic control, a 3-month, cluster randomised controlled trial was conducted in primary healthcare.	The study evaluated T2DM self-management and blood glucose control through Q&As, examinations and interviews, with cost-effectiveness assessed at 12-18 months.	The primary outcome was HbA1c reduction, while the secondary outcomes included changes in self-management behaviour, social cognition, psychology, T2DM skills and health literacy.	Investigators underwent training; respondents were tracked; data were validated; and software issues were addressed to ensure trial accuracy and reliability.	Research leaders should oversee implementation, ensure data accuracy and address AI issues by redesigning the study if necessary to maintain reliability.
**Legends:** MNT = medical nutrition therapy; CVD = Cardiovascular disease; AES-Heart = Australian Eating Survey - Heart version; AI-HEALS = artificial intelligence-based health education accurately linking system; T2DM = type 2 diabetes mellitus; HbA1c = glycated haemoglobin; AI = artificial intelligence							

### Types of DHIs applied in NCD management

Table 2 displays the digital health applications used for managing NCDs, including diabetes, mental health conditions and cardiovascular diseases. It highlights telehealth (n=13), mHealth (n=10) and eHealth (n=2), showcasing technology’s role in PHC for chronic disease management.

**Table 2:** Types of DHIs applied for delivering PHC in NCD management in the WPR.

Service and application type	Study (author and year)
Telehealth: Delivery of healthcare services remotely via video calls, phone consultations or online platforms	Ward et al., 2023; Cross et al., 2022; Imai et al., 2022; Yin et al., 2022; Koh et al., 2023; Tan et al., 2023; Wang et al., 2023; Teo et al., 2023; Andrews et al., 2023; Drabarek et al., 2022; Chew et al., 2023; Teo et al., 2024; Schumacher et al., 2023
mHealth: Use of mobile devices such as smartphones or tablets for health services and information, including applications and SMS-based monitoring	Ward et al., 2023; Cross et al., 2022; Buss et al., 2022; Koh et al., 2023; Teo et al., 2023; Drabarek et al., 2022; Chew et al., 2023; Ju et al., 2022; Teo et al., 2024; Wu et al., 2023
eHealth: Broader digital solutions such as electronic health records, patient portals, online health education and health information systems	Champion et al., 2023; Parker et al., 2022

### Functionality of DHIs

Table 3 highlights diverse DHIs targeting key healthcare stakeholders within the healthcare system, as classified by the WHO^34^: persons with chronic conditions (n=7), healthcare providers (n=2), health system managers (n=1) and data services (n=2), while six studies did not fit these categories, all aiming to improve healthcare delivery.^24,28,29,30,32,33^

**Table 3:** Functionality of DHIs in the WPR according to the WHO classification.

DHI functionality	Study (author and year)
DHIs for persons with chronic conditions	Cross et al., 2022; Imai et al., 2022; Yin et al., 2022; Champion et al., 2023; Koh et al., 2023; Tan et al., 2023; Ward et al., 2023
DHIs for HCPs	Drabarek et al., 2022; Andrews et al., 2023
DHIs for health system managers	Teo et al., 2023
DHIs for data services	Buss et al., 2022; Teo et al., 2024

Legends: DHIs =digital health interventions

### Dimensions of health system challenges

Table 4 outlines the WHO-classified health system challenges addressed by the DHIs. The studies highlighted the following key dimensions: information (n=11), quality (n=4), acceptability (n=14), utilisation (n=3), efficiency (n=12), cost (n=3), accountability (n=3) and equity (n=2), with no focus on availability.

**Table 4:** Dimensions of health system challenges addressed by DHIs according to the WHO classification in NCD management.

Dimension	Study (author and year)	Qualitative analysis
Information	Champion et al., 2023; Imai et al., 2022; Yin et al., 2022; Wang et al., 2023; Andrews et al., 2023; Drabarek et al., 2022; Parker et al., 2022; Ju et al., 2022; Teo et al., 2024; Schumacher et al., 2023; Wu et al., 2023	Digital tools improved data access and sharing, supporting timely decisions (e.g. AI-HEALS for self-management).
Availability	Nil	
Quality	Champion et al., 2023; Andrews et al., 2023; Drabarek et al., 2022; Parker et al., 2022	Tools showed clinical benefits, but concerns included reduced diagnostic accuracy without physical examinations.
Acceptability	Ward et al., 2023; Cross et al., 2022; Champion et al., 2023; Buss et al., 2022; Yin et al., 2022; Koh et al., 2023; Tan et al., 2023; Wang et al., 2023; Teo et al., 2023; Andrews et al., 2023; Chew et al., 2023; Ju et al., 2022; Schumacher et al., 2023; Wu et al., 2023	Users appreciated convenience but raised concerns about privacy, trust and lack of in-person care, especially among less tech-savvy groups.
Utilisation	Cross et al., 2022; Imai et al., 2022; Buss et al., 2022	Use of digital tools varied by age, education and technological skills. Older, educated or urban users engaged more, while others showed low uptake due to access or motivation issues.
Efficiency	Ward et al., 2023; Buss et al., 2022; Yin et al., 2022; Koh et al., 2023; Wang et al., 2023; Ho et al., 2023; Chew et al., 2023; Parker et al., 2022; Ju et al., 2022; Teo et al., 2024; Schumacher et al., 2023; Wu et al., 2023	Digital interventions saved time and reduced clinic burden when well-integrated into workflows.
Cost	Teo et al., 2024; Schumacher et al., 2023; Wu et al., 2023	Some studies noted cost-effectiveness for some tools (e.g. remote blood pressure monitoring), but concerns remained about long-term affordability and technology investment, especially in resource-limited settings.
Accountability	Teo et al., 2023; Drabarek et al., 2022; Chew et al., 2023	Trust issues arose around unclear roles in digital care. Some providers feared losing control or liability in remote settings, and patients were unsure who was responsible for follow-up.
Equity	Cross et al., 2022; Tan et al., 2023	Barriers existed for rural and low-literacy populations, highlighting risks of unequal access and the need for tailored approaches.

## Discussion

The findings highlight the increasing role of DHIs in managing NCDs, particularly within primary care. Telehealth, mHealth and eHealth applications have demonstrated their potential to improve accessibility, efficiency and personalised care across various chronic conditions. Among them, telehealth has emerged as the most widely adopted intervention in the WPR, facilitating remote consultations that closely resemble inperson visits.^16,17,19,21-28,31,32^ The integration of AI into telehealth and mHealth solutions has further enhanced diagnostic precision and patient _engagement._^19,22,27,28,31,33^_However, despite these_ advancements, the ethical and legal dimensions of digital health, especially concerning privacy, liability and regulatory compliance, remain underexplored in many studies. Addressing these gaps is crucial to mitigating potential medicolegal risks associated with digital adoption.

Insights from high-income WPR countries, including Australia, Singapore, China and South Korea, provide valuable guidance for Malaysia’s digital health strategy. Key considerations include optimising user acceptance, ensuring equitable access and strengthening data systems to support evidence-based decision-making.^34^ Established digital health ecosystems emphasise quality control, efficiency and accountability, offering lessons for Malaysia.^35^ The review found that challenges such as weak infrastructure, low digital literacy and limited capacity for continuous service remained major barriers to digital health adoption in primary care, particularly in settings including Malaysia.^36^

The review findings also underscore the potential of wearable technologies for chronic disease management, given the rising adoption of smart devices among Malaysians.^20,25,28,31^ Implementing digital tools such as remote monitoring for hypertension and diabetes management could enhance early detection and patient engagement.^37,38^ Additionally, application-based interventions, behavioural modification programmes and patient-led monitoring approaches present promising avenues for improving health outcomes.^39-41^ However, these initiatives must be tailored to local contexts, addressing cultural preferences, digital readiness and healthcare system constraints.

A key limitation of this review is the limited availability of post-pandemic digital health research in the WPR, restricting region-specific evidence to guide Malaysia’s adoption strategies. This timeframe reflects more sustainable, policy-driven implementations rather than temporary solutions during the COVID-19 pandemic. Additionally, the predominance of studies from high-income settings presents challenges in directly translating findings to Malaysia’s healthcare landscape. Despite these constraints, Malaysia’s Health White Paper (2023) and Digitalization Strategic Plan (2021-2025) demonstrate a commitment to integrating digital health solutions. Future research should focus on implementation strategies, infrastructure development and sustainable financing models to support the successful and equitable adoption of digital health in Malaysia’s primary care, especially in the post-pandemic context, where long-term digital solutions are now being shaped.

## Conclusion

This scoping review highlights the widespread use of digital health, particularly telehealth and mHealth, for managing NCDs in primary care across high-income WPR countries post-pandemic. While these interventions showed positive impacts on efficiency, quality and patient engagement, common challenges included low acceptability among certain groups, digital literacy gaps, infrastructure limitations and inequities in access. Malaysia, as part of the WPR, can draw valuable lessons from these findings by adapting proven strategies to its local context. Future efforts should prioritise context-specific research, inclusive implementation planning and capacity building to support sustainable and equitable integration of digital health in Malaysian primary care.

## Review protocol registration

Hospital Pakar Universiti Sains Malaysia (HPUSM)
